# Short-term outcomes of deeper intubation technique of ileus tube for different types of acute intestinal obstruction patients: A retrospective multicenter study

**DOI:** 10.3389/fonc.2022.1065692

**Published:** 2022-12-22

**Authors:** Yanlu Tan, Fangxu Yin, Zhihua Lu, Peng Huang, Chengcai Zhang, Jiuzheng Sun, Song Wang, Zhensheng Dong

**Affiliations:** ^1^ Department of Interventional Oncology, Central Hospital of Zibo, Zibo, China; ^2^ Department of Thyroid and Breast Surgery, Binzhou Medical University Hospital, Binzhou, China; ^3^ Department of General Surgery, Qilu Hospital (Qingdao), Cheeloo College of Medicine, Shandong University, Qingdao, China; ^4^ Department of Gastrointestinal Surgery, Central Hospital of Zibo, Zibo, China; ^5^ Department of Hepatobiliary Surgery, Central Hospital Affiliated to Shandong First Medical University, Jinan, China; ^6^ Department of Pediatric Surgery, Tianjin Medical University General Hospital, Tianjin, China; ^7^ Department of General Surgery, The Fifth Division Hospital, Xinjiang Production and Construction Corps, Bole, China

**Keywords:** bowel obstruction, ileus tube, deeper intubation technique, short-term outcomes, traditional intubation technique

## Abstract

**Background:**

Our previous research reported a novel deeper intubation technique (DIT) of the ileus tube for acute bowel obstruction patients. The present study was designed to evaluate the effect of this novel technique on the clinical outcomes of patients with obstruction using a large cohort.

**Methods:**

The detailed clinical data were analyzed retrospectively from 496 obstruction patients who underwent intubation technique from 2014 to 2019 in five hospitals. The patients were divided into either the DIT group or the traditional intubation technique (TIT) group. The groups were matched in a 1:1 ratio using propensity scores, and the primary outcome was the short-term clinical outcomes for patients.

**Results:**

The baseline characteristics were similar between the DIT group and the TIT group after matching. Compared with the TIT group, the DIT group had a significantly deeper intubation depth, with shorter hospital days, shorter time to first flatus and defecation, lower pain score, increased drainage volume, and lower emergency surgery rate. Importantly, the inflammatory factors such as white blood cell, C-reactive protein, and procalcitonin levels were significantly lower in the DIT group. In addition, the DIT treatment was significantly useful for adhesive obstruction patients.

**Conclusion:**

The DIT procedure led to better short-term clinical outcomes compared with the TIT procedure, indicating that DIT is a safe and feasible technique for the treatment of intestinal obstruction that is worthy of further popularization and clinical application.

## Introduction

Intestinal obstruction is a common acute abdomen that can be caused by a variety of reasons, which leads to approximately 15% of all emergency visits for acute abdominal pain (1, 2). Adhesive intestinal obstruction accounts for the majority of this disease. Patients with adhesive intestinal obstruction usually present abdominal pain, vomiting, abdominal distension, and exhaust defecation ceasing (3). Acute intestinal obstruction may lead to electrolyte disturbance, intestinal perforation, intestinal necrosis, septic shock, *etc.* (4). Patients with intestinal obstruction may be treated either by conservative therapy or surgical therapy, and the key point for the treatment of this disease is decompressing the gastrointestinal tract effectively as soon as possible (5, 6).

Ileus tubes have been used in the treatment of intestinal obstruction for nearly 100 years (7). Under the guidance of X-ray or electronic gastroenteroscopy, the ileus tubes are usually placed in the jejunum through the pylorus, duodenum, and Treitz ligament (8, 9). However, due to the insufficiency of traditional ileus intubation, for example, as the depth of catheterization continues to increase, the friction between the guide wire and the lumen will also increase significantly, and the tip of the catheter cannot effectively approach the obstruction site. Our previous research reported a novel deeper intubation technique (DIT) that allows the tip of the tube to reach the proximal end of the obstruction, which was effective for the treatment of adhesive intestinal obstruction (10).

The purpose of the present study is to evaluate the safety and effectiveness of this novel DIT procedure. We retrospectively collected from five hospitals the data of 496 subjects that received either the DIT treatment or the traditional intubation technique (TIT) treatment. Then, the short-term clinical outcomes as well as the inflammatory parameters of these subjects were compared.

## Materials and methods

### Patients

This study was designed as a multicenter, retrospective case–control study comparing the short-term clinical outcomes and safety of the DIT procedure and the TIT procedure. The present study investigated 496 hospitalized patients with acute intestinal obstruction in five hospitals from January 2018 to December 2020. The inclusion criteria were as follows (1): the hospitalized patients had acute intestinal obstruction symptoms like nausea and vomiting, abdominal pain, abdominal distension, and exhaust defecation ceasing, (2) the patients were diagnosed with intestinal obstruction by abdominal X-ray plain films and abdominal CT examination, (3) the patients were suitable for conservative treatment, with no severe abdominal pain or persistent abdominal pain, bloody vomiting or bloody stool, asymmetric abdominal distension, respiratory instability, and even shock, peritoneal irritation, and other strangulated intestinal obstruction symptoms, (4) the patients had no contraindications of tube intubation, such as a history of ENT surgery, esophageal disease, *etc.*, and (5) detailed medical records and follow-up information were available.

### The DIT and TIT procedures

The DIT and TIT procedures were performed using the CLINY Ileus Tube suite (Create Medic, Tokyo, Japan) according to the protocol reported previously. All the enrolled patients were given conservative treatment methods as fasting, intravenous nutrition, anti-infection, maintenance of water, electrolyte, and acid–base balance, *etc.* When patients presented with symptoms of severe abdominal pain, distension worsening, tachycardia, hematemesis, hematochezia, peritoneal irritation, isolated swelling, bowel loops, and even shock, timely surgical treatment would be needed. After multidisciplinary discussions, patients who failed to be intubated were treated surgically. The surgeon probes the abdominal cavity to find and remove the obstructed bowel. An anastomosis or ileostomy is performed depending upon the condition of the patient and the contamination involved.

### Outcome measurement

Details on the average intubation depth, the daily drainage of the gastrointestinal decompression tube, the abdominal pain relief rate, the recovery time for anal exhaust defecation, and the length of hospital stay were recorded. The treatment efficiency was defined as a clinical or radiological improvement, relief of abdominal symptoms, decreased drainage volume, and disappearance of air–fluid levels.

The patients’ pain score and defecation situation were monitored and recorded before and every 24 h after intubation using the Numeric Rating Scale (NRS) (11, 12). The severity of complications was analyzed using the Clavien–Dindo classification standard. Data on blood routine, C-reactive protein (CRP), and procalcitonin (PCT) levels were recorded before and every 24 h after intubation. Data concerning intubation-related complications such as catheterization discomfort/pain, electrolyte disturbance, catheter obstruction, catheter shift/falling off, aspiration pneumonia, intestinal hemorrhage, and intestinal perforation were also collected.

### Propensity score matching analysis

We used propensity score matching (PSM) to limit confounders and overcome possible patient selection bias due to the retrospective study design. A regression model was created based on potential variables (age, sex, body mass index, and comorbidity) associated with the selection of treatment. A 1:1 nearest neighbor matching algorithm with an optimal caliper width of 0.2 without replacement was applied to match the propensity scores.

### Statistical analysis

The data in this study were processed by SPSS 26.0 software (IBM, USA). Comparisons between the two groups were performed by Student’s *t*-test for continuous variables and chi-square test or Fisher’s exact test for categorical variables. All statistical tests were two-sided, and *P*-values less than 0.05 were considered statistically significant.

## Results

### Clinical characteristics of patients

All patients had a history of abdominal surgery and presented acute intestinal obstruction symptoms such as abdominal pain, abdominal distension, vomiting, and exhaust defecation ceasing, and all these patients received conservative treatment. According to the depth of intubation, we divided the patients into the DIT group and the TIT group. In order to eliminate the baseline discrepancies, the groups were matched in a 1:1 ratio using propensity scores (13). **Table 1** shows the patient characteristics of the entire (*n* = 496) and propensity score-matched (*n* = 426) cohorts. After PSM, 426 patients were identified, and the clinical characteristics among the two groups were well balanced (all *P >*0.05; **Table 1**).

**Table 1:** Baseline clinical characteristics of patients between the two groups.

	Entire cohort		*P*-value	Propensity score-matched cohort		*P*-value
	Deeper intubation technique (DIT; *n* = 227)	Traditional intubation technique (TIT; *n* = 269)		DIT (*n* = 213)	TIT (*n* = 213)	
Age, year			0.134			0.249
Mean ± SD	59.82 ± 11.74	59.44 ± 10.15		59.61 ± 11.61	59.38 ± 10.46	
Sex, number (%)			0.533			0.914
Male	164 (44.9%)	201 (55.1%)		154 (50.2%)	153 (49.8%)	
Female	63 (48.1%)	68 (51.9%)		59 (49.6%)	60 (50.4%)	
Body mass index, kg/m^2^			0.589			0.466
Mean ± SD	24.81 ± 3.55	24.37 ± 3.33		24.64 ± 3.36	24.71 ± 3.13	
Comorbidity, number (%)						
Coronary heart disease	29 (55.8%)	23 (44.2%)	0.126	20 (48.8%)	21 (51.2%)	0.870
Hypertension	46 (44.9%)	52 (53.1%)	0.795	39 (50.0%)	39 (50.0%)	1.000
Chronic obstructive pulmonary disease	8 (36.4%)	14 (63.6%)	0.365	6 (42.9%)	8 (57.1%)	0.587
Diabetes mellitus	39 (53.4%)	34 (46.6%)	0.155	34 (57.6%)	25 (42.4%)	0.207

### Short-term clinical outcomes after DIT or TIT treatment

**Figure 1:**
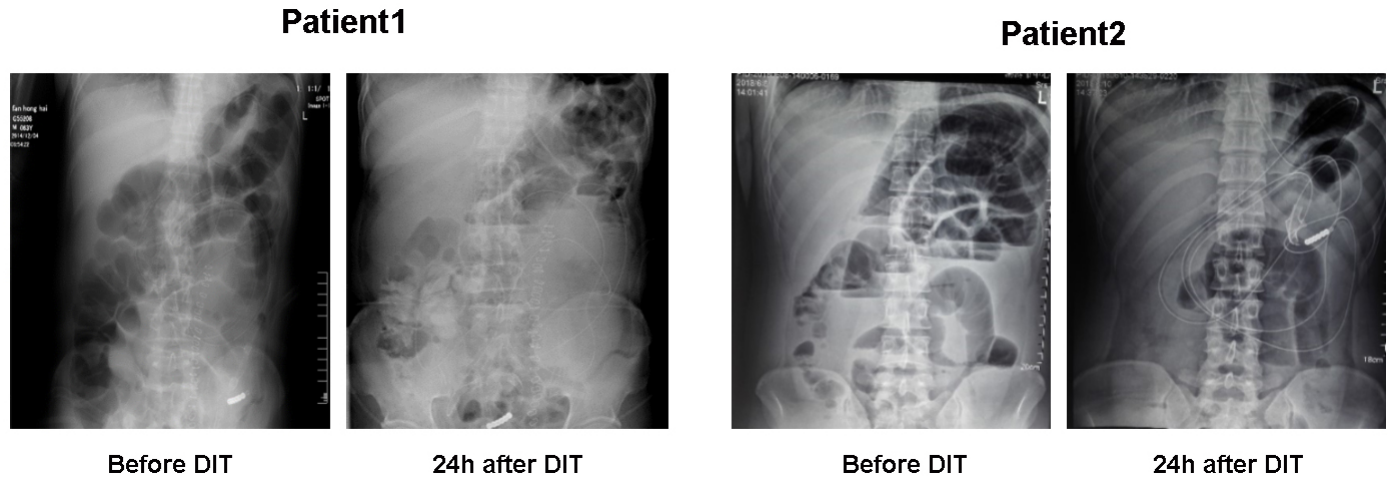
Presentative abdominal X-ray plain films of the patients with acute intestinal obstruction before or 24 h after the deeper intubation technique procedure.

Following the DIT procedure, abdominal radiographs showed an effective therapeutic effect in two example patients with intestinal obstruction (**Figure 1**). Our previous research reported that the intubation could obtain the ileum of the patients in the DIT group, while the TIT treatment could reach the proximal jejunum. As shown in **Table 2**, the DIT group had a significantly deeper intubation depth than the TIT group (221.33 ± 29.12 *vs*. 145.53 ± 21.36 cm, *P* < 0.001).

**Table 2:** Short-term outcomes of the deeper intubation technique (DIT) group and the traditional intubation technique (TIT) group.

Outcome measures	DIT group	TIT group	*P* Value
Intubation depth (cm)	221.33 ± 29.12	145.53 ± 21.36	<0.001*
Numeric Rating Scale (NRS) score before intubation	5.13 ± 1.33	5.06 ± 1.21	0.103
NRS score after 24 h	4.56 ± 2.01	4.89 ± 1.96	0.016*
NRS score after 48 h	3.65 ± 1.21	4.12 ± 1.36	0.002*
First flatus time (day)	1.98 ± 1.12	2.69 ± 1.33	0.036*
Exhaust defecation time (day)	2.56 ± 2.13	3.62 ± 2.51	0.023*
Defecation recovery rate in 24 h	18.8% (40/213)	11.3% (24/213)	0.041*
Defecation recovery rate in 48 h	45.1% (96/213)	29.1% (62/213)	0.001*
Defecation recovery rate 7 days later	83.6% (178/213)	79.3% (169/213)	0.319
Drainage in 24 h (ml)	1,136.25 ± 663.32	796.52 ± 559.61	0.002*
Emergency surgery rate	4.7% (10/213)	8.9% (19/213)	0.125
Hospital stay (day)	8.16 ± 4.31	9.53 ± 5.26	0.016*

*P < 0.05.

Before intubation, there was no difference in the NRS score between the DIT group and the TIT group (5.13 ± 1.33 *vs*. 5.06 ± 1.21, *P* = 0.103), while after intubation, the NRS score in the DIT group was significantly lower than that in the TIT group (24 h after intubation: 4.56 ± 2.01 *vs*. 4.89 ± 1.96, *P* = 0.016; 48 h after intubation: 3.65 ± 1.21 *vs*. 4.12 ± 1.36, *P* = 0.002).

With respect to remission of the disease, the study showed that both the first flatus time and exhaust defecation time were markedly shortened in the DIT group compared with the TIT group (first flatus time: 1.98 ± 1.12 *vs*. 2.69 ± 1.33, *P* = 0.036; exhaust defecation time: 2.56 ± 2.13 *vs*. 3.62 ± 2.51 days, *P* = 0.023). The defecation rate within 24 h (18.8% *vs*. 11.3%, *P* = 0.041) and 48 h (45.1% *vs*. 29.1%, *P* = 0.001) after intubation was significantly increased in the DIT group. Most of the patients in both the DIT group and the TIT group recovered defecation within 1 week after intubation (83.6% *vs*. 79.3%, *P* = 0.319).

Within 24 h after intubation, the drainage volume in the DIT group was higher than that in the TIT group (1,136.25 ± 663.32 ml *vs*. 796.52 ± 559.61 ml, *P* = 0.002). Although the emergency surgery rate was lower in the DIT group, the difference was of no significance (4.7% *vs*. 8.9%, *P* = 0.122), and patients in the DIT group had a shortened hospital stay (8.16 ± 4.31 *vs*. 9.53 ± 5.26, *P* = 0.016). As for intubation-related complications, there was no significant difference between the two groups (12.2% *vs*. 13.6%, *P* = 0.775), and according to the Clavien–Dindo classification, the two groups also showed no significant difference regarding the complication grade (**Table 3**).

**Table 3:** Detailed overview of the complications.

Complication rate (Clavien–Dindo classification)	Deeper intubation technique group	Traditional intubation technique group	*P*-value
Overall	12.2% (26/213)	13.6% (29/213)	0.775
I–II	6.6% (14/213)	3.8% (8/213)	0.275
III	4.7% (10/213)	8.9%(19/213)	0.125
IV	0.9% (2/213)	0.9% (2/213)	1.000

### Efficacy of the treatment for different types of intestinal obstruction

To further evaluate the effect of DIT procedure in the treatment of different types of intestinal obstruction, we analyzed the data according to the etiology of obstruction and divided the patients into three groups: adhesive obstruction, fecal obstruction, and cancerous obstruction. As shown in **Table 4**, there were 96 patients diagnosed with adhesive intestinal obstruction, and 88 (91.7%) of them recovered after the DIT treatment. Meanwhile, in the TIT group, 99 patients were diagnosed with adhesive intestinal obstruction, and 80 (80.8%) patients showed adequate recuperation (*P* = 0.037). In accordance with our previous research, no significant difference was shown between the two groups when it comes to fecal obstruction (*P* = 0.538) and cancerous obstruction (*P* = 0.328).

**Table 4:** Therapeutic efficacies for different types of intestinal obstruction.

Overall efficacy (effective/ineffective)	Deeper intubation technique group		Traditional intubation technique group		*P*-value
	Effective	Invalid	Effective	Invalid	
Adhesive obstruction (*n* = 187)	88	8	80	19	0.037*
Fecal obstruction (*n* = 148)	62	13	57	16	0.538
Cancerous obstruction (*n* = 83)	28	14	32	9	0.328

*P < 0.05.

### Inflammatory indexes after DIT or TIT treatment

**Figure 2:**
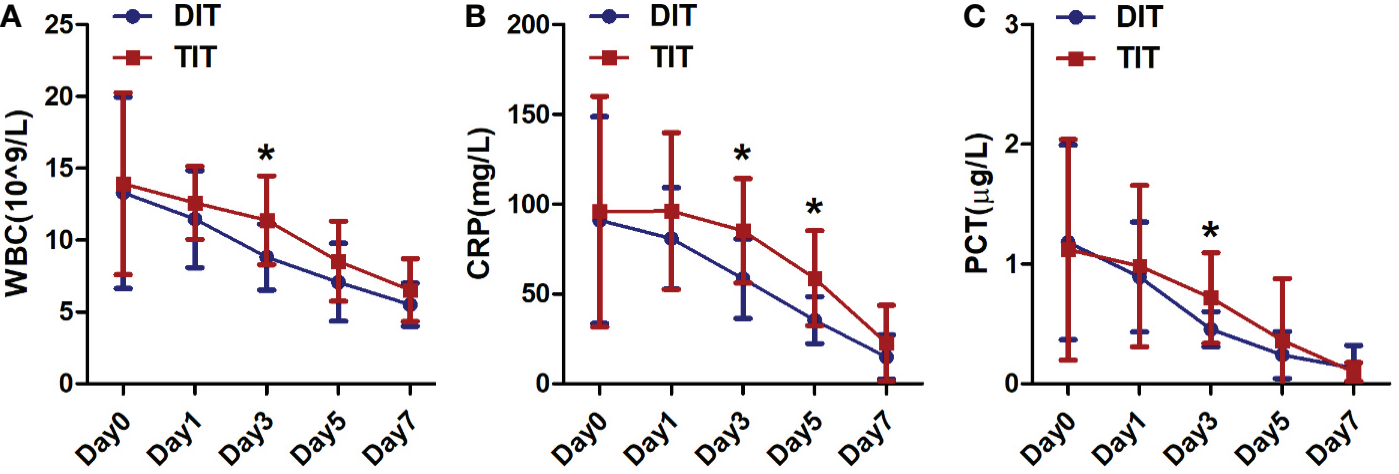
Comparison of levels of certain inflammatory indexes in the traditional intubation technique group and the deeper intubation technique group. **(A)** White blood cell count, **(B)** C-reactive protein, and **(C)** procalcitonin. * P<0.05.

Inflammatory indexes such as the WBC, CRP, and PCT were used to evaluate the level of infection and remission of the disease. As shown in **Figure 2**, the WBC, CRP, and PCT levels were of no significant difference between the two groups before intubation. The WBC, CRP, and PCT levels were markedly lower in the DIT group compared with the TIT group 3 days after intubation, while the difference between the two groups gradually narrowed and the levels of these indicators decreased by degrees with prolonged intubation time, accompanied by the remission of the disease.

## Discussion

Intestinal obstruction is one of the most common reasons of all emergency department visits for acute abdominal pain (1). Serious complications such as septic shock, intestinal perforation, severe electrolyte disturbance, and multiple organ dysfunction syndrome may occur, which may lead to approximately 5%–10% death rate (14). The treatment strategies for acute intestinal obstruction are relatively complex, which could be mainly divided into surgery treatment and conservative treatment (3, 15). The key point for the treatment of this disease is adequate drainage of gastrointestinal contents and reduction of gastrointestinal stress effectively (16). After exclusion of strangulated intestinal obstruction, gastrointestinal decompression is the most effective strategy for conservative treatment.

For the TIT procedure, the diameter of the matching guide wire of the intestinal obstruction catheter is 0.045 cm with high hardness, and the friction between the catheter and the guide wire is large, so it is difficult to control the catheter and the guide wire (10). Moreover, due to the early application of analgesics and somatostatin in patients with intestinal obstruction, infection and electrolyte imbalance may occur, which affect intestinal peristalsis (17). As a consequence, the traditional intubation technique could only decompress the stomach and the proximal jejunum, which might be effective for high intestinal obstruction, but the effect is usually limited for low bowel obstruction.

Our previous research presented a novel DIT procedure. Using the CLINY Ileus Tube suite, a zebra guide wire with a diameter of 0.035 with ideal flexibility was selected, which made it easier to control the catheter, and the catheter was as close to the obstructed site as possible without the aid of intestinal peristalsis. Moreover, digital gastrointestinal fluoroscopy technology can not only expose and collect images in real time, realize intermittent operation, and avoid X-ray radiation of the operator but also allow patients to change position at any time. Furthermore, when intubation encounters obstacles such as intestinal folds or swerve, an appropriate volume of air could be injected into the bowel *via* the catheter to change the stereo direction of the bowel, changing the patients’ posture and guiding the weighted tip to conform to the track of the bowel at the same time. In addition, water-soluble iodine contrast medium could also be injected into the bowl to observe the stereo track of the bowel and stimulate peristalsis. Therefore, we could effectively forward the tip of the tube closer to the obstruction site and decompress the intestinal contents more sufficiently.

To confirm the safety and effectiveness of this procedure, we conducted a multicenter, retrospective cohort study comprising 496 subjects. Although there were no significant differences in baseline characteristics between the two groups, we still tried to minimize potential imbalances, such as in the PSM analysis. The two groups were matched in a 1:1 ratio using propensity scores, and after matching, the two groups were comparable. The present study showed that the intubation depth in the DIT group was significantly deeper than that in the TIT group. As is known to all, intestinal peristalsis is the main power forwarding the tube in the TIT group, and patients usually present weakened or even vanished intestinal peristalsis due to the application of analgesics, abdominal (intestinal) infection, and electrolyte disorders (18). The deeper intubation led to a more effective decompression, and an increased drainage volume was obtained in the DIT group. As a consequence, the symptoms of the patients were relieved faster. As time went by, the tube moved forward, and the tip of the tube in TIT group might also reach the same site as the DIT group finally, so the advantage of DIT in the early stage of acute intestinal obstruction is more evident. The DIT procedure can relieve the patient’s abdominal pain and ensure the rapid recovery of the patient’s intestinal function. This may be related to the reduction of intestinal pressure and the rapid restoration of the blood supply capacity of the intestinal wall (19, 20).

It is worth noting that the emergency surgery rate was lower in the DIT group, although the difference was of no significance. The tendency may also benefit patients with acute intestinal obstruction a lot. The shortened hospital stay reduces the burdens of the patients’ family and leads to better social economic benefits. Moreover, intubation-related complications such as catheterization discomfort/pain, catheter obstruction, catheter shift/falling off, aspiration pneumonia, and intestinal hemorrhage/perforation were of no significant difference between the two groups. All the aforementioned results indicated that the DIT procedure was a safe and effective treatment strategy for patients with acute intestinal obstruction.

The effect of DIT on different types of intestinal obstruction was also investigated. Consistent with our previous research, the overall efficacy of the DIT procedure was up to 91.7% for adhesive intestinal obstruction, the most common type of intestinal obstruction (21, 22). This was significantly more effective than that in the TIT group. This is important for patients who had a history of abdominal surgery, as surgery is quite challenging and risky, and the complication rate is high for these patients (23). Moreover, surgery may enhance the ankylenteron and induce the recurrence of intestinal obstruction over and over again (24). Although no significant difference was shown when it comes to fecal obstruction, the overall efficacy was still better in the DIT group compared with the TIT group. For patients with fecal obstruction, a certain aerogenic agent (mainly composed of citric acid and sodium bicarbonate) or liquid paraffin would be given through the tube, which could facilitate the dissolution and cure of stercolith (25). There was no significant difference in the efficacy of the two methods for patients with malignant obstruction, and many patients who suffered from cancerous obstruction usually received surgery.

Importantly, to evaluate the effect of the DIT procedure, the study also analyzed the inflammatory indexes such as WBC, CRP, and PCT, as these indicators could simply and practicably reflect the severity of the disease, the degree of inflammatory responses, and the infectious condition (26, 27). Thus, these indicators were of vital importance for patients with acute intestinal obstruction. The present study showed that the DIT procedure could decrease these inflammatory indexes rapidly and effectively, accompanied with the relieved symptoms and faster recovery.

The highlight of this study mainly relies on the large sample size and the multicenter design. Moreover, related data were matched using propensity scores to eliminate the base discrepancies, which made the results more rigorous. Compared with our previous research, the present study proved that the DIT procedure is more convincingly a safe and effective treatment strategy for acute intestinal obstruction. Of course, long-term survival data is warranted in a further study. Based on the aforementioned results, a multicenter randomized controlled trial would be welcome and provide higher-level data of evidence-based medicine.

In conclusion, the DIT procedure is a safe and effective treatment strategy for acute intestinal obstruction. Compared with the TIT procedure, the DIT procedure showed better short-term clinical outcomes and decreased the inflammatory responses more effectively, which is worthy of clinical application and popularization.

## Data availability statement

The raw data supporting the conclusions of this article will be made available by the authors, without undue reservation.

## Ethics statement

The studies involving human participants were reviewed and approved by The Clinical Ethical Committee of Central Hospital of Zibo. The patients/participants provided their written informed consent to participate in this study. Written informed consent was obtained from the individual(s) for the publication of any potentially identifiable images or data included in this article.

## Author contributions

ZD and SW designed the research. YT, FY, and ZL were involved in data acquisition, analysis, and interpretation. YT wrote the paper. PH and CZ contributed to make the tables and figures. JS revised the paper. All authors contributed to the article and approved the submitted version.
